# A high-resolution spatiotemporal wildfire propagation dataset for the Mediterranean and Europe

**DOI:** 10.1038/s41597-026-06965-2

**Published:** 2026-03-11

**Authors:** Simon Müller, Anja Hofmann-Böllinghaus, Zhimin Chen, Kristin Vogel, Philipp Benner

**Affiliations:** 1 Bundesanstalt für Materialforschung und -prüfung (BAM), Unter den Eichen 87, 12205 Berlin, Germany; 2 Technische Universität Berlin, Straße des 17. Juni 135, 10623 Berlin, Germany

**Keywords:** Natural hazards, Environmental impact

## Abstract

Wildfires are becoming more frequent and severe under the influence of climate change, posing increasing risks to ecosystems, human health, and infrastructure. Accurate spatiotemporal data on wildfire propagation is essential for advancing fire behavior modeling, improving management strategies, and mitigating future impacts. However, existing datasets with both high spatial and temporal resolution are rare, costly, and time-consuming to produce. To address this gap, we present FireSpread_MedEU, a dataset comprising 320 consecutive burned area maps from 103 wildfire events across the Mediterranean and Europe between 2017 and 2023. Burned areas were derived from high-resolution Planet optical satellite imagery (~3 m spatial, mostly daily temporal resolution) using a semi-automated workflow, followed by manual refinement to ensure highest accuracy. Each dataset entry is enriched with detailed metadata and a subjective quality assessment. With its high level of spatiotemporal precision, FireSpread_MedEU provides essential data for the development and validation of machine learning models or wildfire simulation models. It opens new research opportunities in wildfire behavior analysis, risk assessment, and predictive modeling.

## Background and Summary

In populated regions, wildfires, which are a natural part of many ecosystems, pose a significant threat to the economy, infrastructure, and human health^1^. As rising temperatures and prolonged droughts continue, studies predict that ongoing climate change will increase the likelihood, duration, and size of future wildfires^2–4^. As a result, appropriate wildfire management strategies are important, and modelling wildfire behavior can be one important tool to mitigate future impact. For their calibration and evaluation, accurate burned area maps with high spatial and temporal accuracy are crucial.

Mapping burned areas is an ongoing field of wildfire research^5,6^, and oftentimes, burned areas are automatically identified from satellite images after an event ended (e.g.^7,8^). Non-commercial satellites allow high spatial resolutions (Sentinel-2 and Landsat with up to 10 m), but their revisit time of multiple days limits the temporal resolution of a burned area mapping. As a result, independent of the geographical region, most available burned area datasets with high spatial resolution are temporally static, including only the final burned areas (e.g.^9–12^).

While regional and global datasets with regular temporal burned area propagations do exist (e.g.^13–15^), they mostly rely on the same data products derived from MODIS or VIIRS sensors^16,17^. This limits their spatial accuracy, since the original products have spatial resolutions of 500 m and 375 m, respectively. Additionally, spatial uncertainties due to mixed pixels or small patches are observed, and temporal deviations of multiple days to MODIS active fire detections are known to exist^18,19^. Openly available wildfire propagation data with both, high spatial and high temporal resolution are sparse and either recorded by ground-based mapping, airplanes, or Unmanned Ariel Vehicles (UAV) (e.g.^20–22^). Since tracking wildfire propagation over multiple days with high accuracies in space and time is time consuming and expensive, it is rarely done. As a result, very few high-resolution datasets for spatiotemporal wildfire propagation exist (e.g.^22^). Table 1 shows a list of recent datasets that track fire progression in space and time. For every dataset, different traits like the geographical extend, the original data source, spatial and temporal resolution, and individual limitations are provided.

**Table 1:** List of recent datasets that track the spatiotemporal fire progression in different geographical areas.

Product Name	Study	Propagation based on	Region	Time period	Fire instances	Spatial Res.	Temp. Resolution	Limitations
GlobFire	Artes *et al*.^14^	MODIS burnt area product MCD64A1^19^	Global	2000–2021	Bound to MCD64A1	500 m	Daily	Spatial and temporal accuracy is limited to the underlying MODIS product.
Fire Atlas	Andela *et al*.^13^	MODIS burnt area product MCD64A1^19^	Global	2003-ongoing	Bound to MCD64A1	500 m	Daily	Spatial and temporal accuracy is limited to the underlying MODIS product.
FIRED	Balch *et al*.^15^	MODIS burnt area product MCD64A1^19^	United States	2001–2019	51,871	500 m	Daily	Spatial and temporal accuracy is limited to the underlying MODIS product.
FEDS	Chen *et al*.^30^	VIIRS active fire product^31^	Californa	2012–2020	35,337	375 m	Half-daily	Inaccuracies in final burned area possible.
PT-FireSprd	Benali *et al*.^22^	Manual reconstruction from Satellites, UAV, airborne, field observations	Portugal	2015–2024	155	<1 m to 4000 m	Up to Multi-daily	Only large fires included (>100 ha).
CFSDS	Barber *et al*.^32^	NBAC, MODIS and VIIRS active fire product	Canada	2002–2021	3,269	180 m	Daily	Only very large fires included (>1000 ha).
FireSpread_MedEU	This study	Planet Labs Inc. and manual optical refinment	Mediterranean and EU	2017–2023	103	3 m	Daily	Only limited amount of bands available.

To address the displayed gap of available wildfire propagation data with high spatiotemporal accuracy (Table 1), we present FireSpread_MedEU^23^. This dataset includes the individual propagation steps of 103 wildfire events in Europe and the Mediterranean with high spatial (~3 m) and high temporal (at best daily) resolution. For its creation, we sampled 103 wildfire events from the European Forest Fire Information System (EFFIS) burned area database^24,25^ and created propagating burned area maps for the complete duration of each sampled wildfire. The sampling process is described in detail in the Methods section. The EFFIS burned area database refers to their real-time updated Burnt Areas database, which includes final burned areas for 40 countries in the geographical extend (24.75°W-45°E, 27°N-72°N). Despite indicated by EFFIS name, the Burnt Areas database does not only include forests, but all kinds of vegetational fires. These are mapped in a semi-automatic procedure, and after visual verification, the final burned areas are derived using MODIS, Sentinel-2, and VIIRS satellite data^24^.

To create the propagating burned area maps for each sampled fire, optical satellite data with high spatial and temporal resolution from the commercial satellite operator Planet Labs Inc. were used. The burned areas were extracted semi-automatically from the satellite images. To ensure highest possible accuracy, all instances were checked and refined manually. While many wildfire events have daily updated propagation steps, extensive smoke, clouds or (partly) missing observations prevented visual analysis for individual days. To still be able to fully reproduce the wildfire propagation from FireSpread_MedEU, these missing instances are provided without a burned area polygon, and the last available pre-fire date prior to each wildfire is included as well. In total, 649 individual entries for 103 fires with average lengths of 5.3 days were collected in a single shapefile and enriched with metadata and descriptive information as well as a subjective quality assessment by the authors. Excluding the missing and pre-fire dates, individual burned areas are available for 320 propagation steps.

The code for the semi-automatic process is uploaded on GitHub and can be adapted to other satellite images with different resolutions: https://github.com/BAMresearch/wildfire_prop_database.

## Methods

### Data sources

The burned areas in FireSpread_MedEU were derived from Dove Classic (PS2) and SuperDove (PSB.SD) CubeSat images from the commercial satellite operator Planet Labs Inc. The satellites carry four framed imagers with butcher-block filters, providing images with 3 to 4.1 m resolution, daily revisiting times, and global coverage. Where possible, we used surface reflectance data from the four channels blue (455–515 nm), green (500–590 nm), red (590–680 nm), and near infrared (780–885 nm)^26^. In rare cases, only Top of Atmosphere radiance was available. Data access was provided by the European Space Agency (ESA) through their Third Party Missions scheme.

The employed CubeSats produce optical images with high spatial and temporal resolution. In the absence of clouds or thick smoke, fire propagation of distinct wildfire events can be tracked over multiple days for their entire duration. If a clear fire front was observed for consecutive revisits of the satellites, the wildfire event was considered suitable, and a respective satellite image was downloaded for every day that the fire front changed visibly. Individual days can experience heavy smoke development, large cloud cover or (partly) missing satellite coverage. In such cases, propagation could not be monitored daily, sometimes skipping one or multiple days. To provide information on all fire dates of every event, these instances are still included in the dataset but instead of a burned area polygon, the reason for its absence is provided. Overall, 103 individual fire events were identified. For these events, FireSpread_MedEU includes burned areas for 320 distinct fire propagation steps, starting with the last available pre-fire image and ending with the fully grown burned area.

To find suitable wildfire events, i.e., events for which burned area development could be tracked over multiple days, the real-time updated Burnt Areas database was downloaded from the EFFIS website (https://forest-fire.emergency.copernicus.eu/applications/data-and-services) and filtered by length (a duration of at least two days), size (larger than 100 ha), and year (2017 onwards, to assure Planet coverage). The remaining events were scanned randomly in the Planet explorer, looking for instances with visibly growing fire fronts over a period of at least two days and minimal obstruction from smoke, clouds or a fire growth beneath the tree cover. If an event was found, all available Planet images were downloaded for the selected area and the selected dates. The selected areas were often divided over multiple swath paths of the Planet satellites, which were downloaded individually. Afterwards, the swath paths were merged in Python (version 3.11.9), creating a single Planet image for every day with suitable images. The merging process was fully automated and scales well for multiple images and fire instances.

Every entry in the dataset was enriched with additional information that is either based on the satellite instruments metadata, or on subjective assessments from the authors. A detailed description is given below.

### Semi-automatic burned area extraction and manual refinement

To create the date related burned area polygons, the merged images were processed semi-automatically. Before an image was used for burned area selection, it was stretched according to its band specific intensity values and scaled to values between 0 and 255. For the stretching, the 2^nd^ and the 98^th^ percentile were chosen as cutoff values, and every pixel below or above these values was set to the new respective minimum or maximum. Afterwards, min-max normalization was performed. The result was multiplied by 255 to scale all values to a range between 0 and 255 for better visual representation and smoothed with a uniform filter (pixel size of five) to decrease the noise in the individual pixels.

This pre-processing step removes extreme and potentially faulty values and ensures a similar optical appearance of the investigated images. From the pre-processed images, the burned areas were separated based on an individually adapted threshold of the near infrared (NIR) band. Individual thresholds are necessary, since differences in vegetation, and the presence of smoke or clouds can impact the pixel intensity values of the NIR band. Applied threshold values ranged from 70 to 150 and were chosen based on expert judgement after optical comparisons with different band combinations of the original image. Thresholding creates a binary raster, in which all pixels below the defined NIR value are set to 1. This process leads to unavoidable false positive/false negative pixels, since not all burned pixels are equally affected by the fire, and since other land cover classes can show small NIR values as well (e.g., waterbodies). To remove false positive pixels (i.e. non-burned pixels with low NIR values), the multidimensional image processing package of SciPy^27^ was used to combine adjacent pixels below the threshold into uniquely labeled clusters. Then, non-burned clusters were removed. The remaining burned area clusters often still contained patches that either really belonged to unburned holes surrounded by burned surface, or to false negative pixels that are burned but display too high NIR values to be included in the thresholding process. Since we did not find a (computationally feasible) method to integrate these false negative patches into the clusters, while also keeping the unburned holes, we chose to only include the external boundary of each burned area to our dataset.

To transform the burned area clusters from a raster image into a GIS readable polygon, alpha shape calculations were applied. With the Alpha Shape Toolbox^28^, a concave hull (i.e. the alpha shape) was calculated around every burned area pixel in the raster, creating the final polygon. The resulting polygon was then saved in the shapefile format. The complete workflow is illustrated for one image on GitHub (https://github.com/BAMresearch/wildfire_prop_database).

### Quality assessment

Quality assessment was performed subjectively based on the authors expertise. Thereto, four quality parameters were defined: low, medium, high, and very high. Very high quality is only given to burned area observations in images without cloud occurences or non-obscuring smoke. In high quality images, clouds or smoke can be present, but the outline of the burned area must still be distinguishable clearly. Medium quality is given to images in which clouds or smoke partly cover the burned area, but accurate border detection is mostly still possible. Burned areas with a low quality label are partly obstructed by clouds or smoke, preventing a reliable detection of the burned area border, even using the NIR band. The quality assessment was performed for all images by three to four authors independently. In cases of discrepancy, the final quality was determined by majority voting. In case of ties, the lower quality rating was used.

**Fig. 1:**
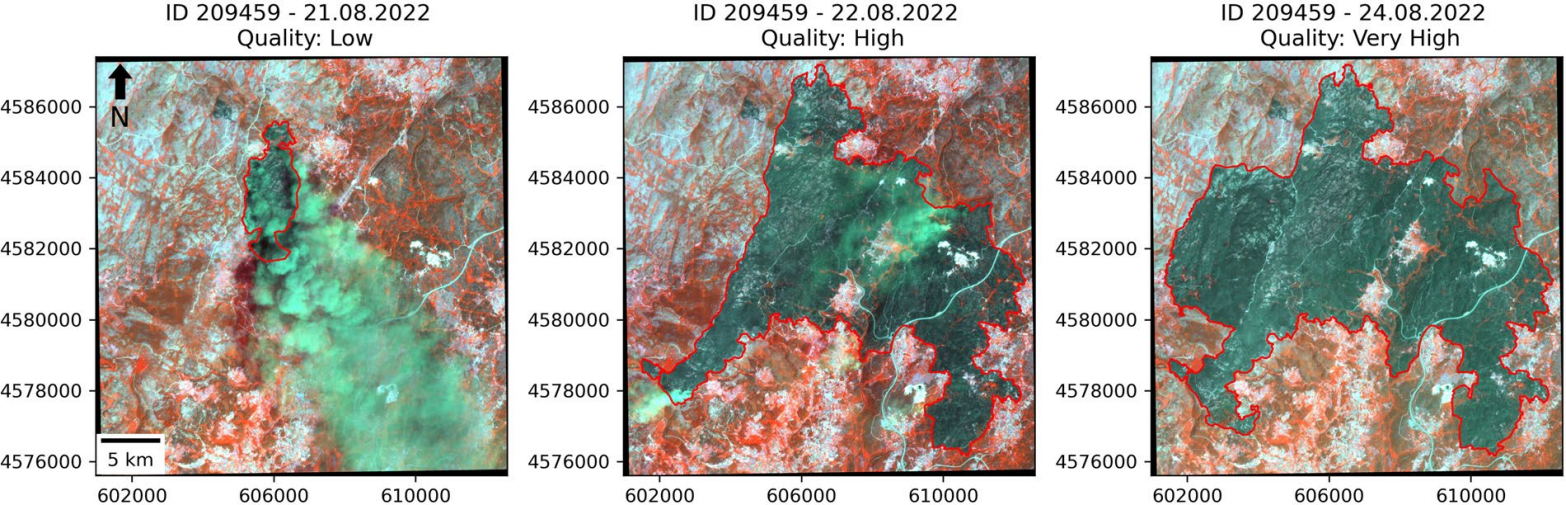
Examples for low, high, and very high-quality labels. The three images show consecutive propagation steps from one of the 103 wildfire events, the respective ID and the propagation dates are shown in the title. The small black spot north-west of the fire outline is a different fire with a distinct ID. Imagery © 2025 Planet Labs Inc.

**Fig. 2:**
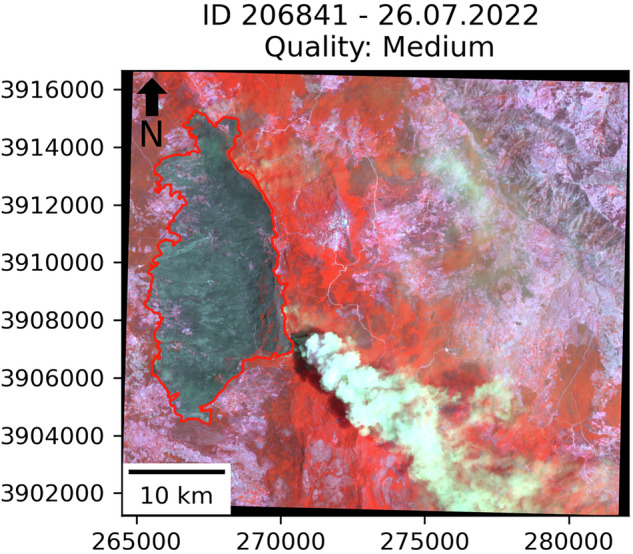
Example for a medium quality label. The image shows one propagation step of a specific wildfire event, the respective ID and the propagation date is shown in the title. Despite the smoke, most of the outline is still traceable with high spatial accuracy. Imagery © 2025 Planet Labs Inc.

To illustrate examples for the different labels of the quality assessment, Fig. 1 shows three propagation steps of the same fire. Every step has a different quality label, displaying examples for low, high, and very high quality. Figure 2 shows the burned area of a different wildfire event, which was labeled as medium quality. The burned area boundaries are highlighted in red. NIR, green, and red band of the employed CubeSats were used as the respective RGB channels in all images. Thereby, the burned area is visible through the dark color, clearly distinguishable from the surroundings.

In Fig. 1, thick smoke is seen in the low-quality image, covering large areas in the south-east of the burned area. Despite the presence of smoke, a precise border can be drawn in the north-western part of the burned area, where the ascending smoke is blown away by the wind, opening the view for the area underneath. Nevertheless, no accurate prediction can be made for the large area directly below the smoke plume, resulting in the low-quality label. Although the near-infrared channel onboard the Planet CubeSats can penetrate smoke and clouds to some extent, thick instances still prevent a clear view of the burned area. In the high-quality image, small smoke plumes are visible in the north-east and south-west of the burned area. Especially in the north-east, the smoke plume is thin and transparent. As a result, it can be penetrated by the NIR band, allowing accurate predictions of the burned area underneath. In the south-west, the smoke plume is thicker, but the outline can still be followed with high certainty. The image with very high quality does not include smoke or clouds and the outline of the burned area is perfectly distinguishable from the non-burned surrounding.

Figure 2 displays an example for the medium quality label. The fire front is producing smoke plumes in the eastern part of the burned area, but the wind direction still allows to identify its outline with high accuracy. Only in the small area directly beneath the thick smoke plume in the south-east, the border of the burned area is not clearly traceable. Since the vast majority of the burned area is still accurately described, the image received the medium quality label.

### Surface information

Surface information is an important factor in wildfire propagation, and we therefore added land cover classes developed by the United Nations Food and Agriculture Organization’s (UN FAO) Land Cover Classification System (LCCS)^29^ to every polygon. The respective land cover classes are derived from multiple satellite sources and provided yearly. We downloaded maps for all years from 2017 onwards from the Copernicus Climate Data Store (https://cds.climate.copernicus.eu/datasets/satellite-land-cover?tab=overview) and clipped every burned area polygon of the dataset to the land cover map of its respective fire year. At the time of dataset creation, land cover maps until 2022 were available. For the two events of 2023, the reference map of 2022 was used. After clipping, the fractional coverage of each land cover class was calculated and added to the respective dataset entry. To facilitate its use, classes of similar land cover were combined into a single class (e.g., all individual cropland classes were merged into a single class cropland). No surface information is provided for the pre-fire date or for missing burned area instances, due to the lack of a burned area polygon.

## Data Records

The dataset is available at Zenodos data repository (https://zenodo.org/records/18200075)^23^. The data comprise a shapefile named FireSpread_MedEU, as well as a PDF file called feature_description.pdf. The shapefile includes the polygons of every available burn date of every wildfire event, as well as the last available pre-fire date. It also includes all fire dates, for which no polygon could be provided. The reason for a missing burned area polygon is given in the Info variable of the dataset and can include (partly) missing planet imagery, cloud or smoke obstruction, or a stagnating fire front.

The specific step of burned area propagation is provided through the Prop_step variable. A Prop_step number is only given to entries with burned areas, a value of 0 corresponds to the last available pre-fire date. The first observable burned area of every wildfire event therefore starts with Prop_step number 1. Following burned areas of the same fire event have increasing propagation numbers, with step sizes varying from one to multiple days, depending on the availability of usable images. Daily coverage is the best possible option and in line with the revisiting times of the Planet satellites.

Every entry of the dataset includes additional variables, which are shown and explained in Table 2. The same description is also provided in the feature_description.pdf.

**Table 2:** Variable names of the final fire propagation database and their description.

Variable name	Description
EFFIS_id	Id of the same burned area in the EFFIS Burnt Areas database^a)^.
Prop_step	Propagation step number of a burned area instance. Step size varies from one to multiple days, depending on cloud/smoke obstruction and image availability. Last available pre-fire date has a Prop_step of 0.
Acqu_date	Year, month, and day of Planet image acquisition (YYYY-MM-DD).
Acqu_time	Exact time of Planet image acquisition (hh:mm:ss).
BA (ha)	Size of the burned area at the specific propagation step in ha.
Quality	Subjective quality assessment of the authors. Quality labels are low, medium, high, and very high.
Clouds	Binary identifier for cloud obstruction: 0 = no clouds; 1 = clouds obscure vision of the burned area.
Smoke	Multiclass identifier for smoke obstruction: 0 = no smoke; 1 = light smoke; 2 = medium smoke; 3 = heavy smoke.
Info	Additional information for the distinct burned area. Reasons for missing burn dates are provided here. Possible reasons area: planet image missing, planet image partly missing, image too cloudy, image too much smoke, no change in burned area.
geometry	Shapefile geometry of the burned area. Empty for missing burn dates.
Crop	Fraction of cropland, as classified by the LCCS
Crop/Veg	Fraction of cropland and vegetation mixtures, as classified by the LCCS
Tree-Broad	Fraction of broadleaved tree cover, as classified by the LCCS
Tree-Needl	Fraction of needle leaved tree cover, as classified by the LCCS
Tree-Mixed	Fraction of mixed tree cover, as classified by the LCCS
Veg-Mixed	Fraction of vegetational mixtures, as classified by the LCCS
Shrub	Fraction of shrubland, as classified by the LCCS
Sparse Veg	Fraction of sparse vegetation, as classified by the LCCS
Urban	Fraction of urban areas, as classified by the LCCS
Bare	Fraction of bare areas, as classified by the LCCS
Grassland	Fraction of grassland, as classified by the LCCS

^a)^Download real-time updated Burnt Areas database at https://forest-fire.emergency.copernicus.eu/applications/data-and-services (last accessed 11.12.2025).

## Technical Validation

The created polygons were manually validated and, where necessary, refined using the open-source software QGIS (Version 3.32.0). Thereby, the created burned areas were optically compared to their respective images, using different combinations of all available bands. Offsets and mismatches induced by clouds, smoke or the alpha shape algorithm were corrected and aligned according to the optically distinguishable burned area.

## Usage Information

Individual wildfire events can be selected according to their unique Effis_id. As indicated by the name, the Effis_id in FireSpread_MedEU is identical to the one in the EFFIS burned area database, simplifying interoperability between the two datasets. For each event, every fire date is given through the Acqu_date variable, also including dates for which no burned area could be extracted. The reason for missing burned areas is specified in the Info variable of the respective entry.

Visible fire progression can easily be derived from the Prop_step attribute. At every propagation step, the geometry of the respective burned area is given. The pre-fire instance has a Prop_step of 0, but no burned area can be provided. To still be able to calculate the fire growth to the first propagation step, a very small, point-like polygon is provided at the center of the first burned area polygon of the respective fire. To investigate the fire growth over time, the geometry from the previous propagation step must be subtracted. Although this growth can be used as an indicator for the speed of spread, it is important to notice that fire spread changes drastically over the course of 24 hours. This prevents the derivation of a conclusive fire spread parameter with the FireSpread_MedEU data. The number of days between two propagation steps can be retrieved either by calculating the difference between the dates, or by summarizing all days without a burned area polygon between the two investigated propagation steps. The burned area with the highest propagation step indicates the final burned area of an event, after which no propagation could be observed.

Due to missing images or visual obstructions, several fire events include observation gaps of one to multiple days between individual instances of fire propagation. Depending on the use-case of this dataset, this can present a limitation and there are different options of how to handle such gaps. Since we solely focused on Planet satellites to offer best spatial resolution, missing dates can potentially be filled through other satellite sources with different sensors. Especially shortwave infrared (SWIR) sensors or non-optical technologies like Synthetic Aperture Radar (SAR) can help to penetrate thick clouds or smoke^5^ and thereby potentially increase the temporal coverage of available burned area dates. By using the Info column, our dataset can be filtered for reasons for observation gaps and, where applicable, other sources can be employed to fill the missing dates.

One potential application of the dataset includes the validation of fire spread models. Here smallest possible differences in time and day are important to ensure accurate modelling, and fire steps for which daily propagating burned areas are available are of highest interest. For long wildfire events, instances of daily updated burned areas and gaps of multiple days can often both be observed. Here, the individual steps with daily propagation can still prove valuable. Alternatively, 32 fire events do not include any missing fire dates.

Since our dataset provides very high spatial and temporal resolution, another use-case includes the validation of burned areas derived from other sources (e.g., to validate automated burned area detection algorithms for satellites). In such cases, the dataset needs to be filtered for the best matching fire date and time, using the Acqu_date and Acqu_time variable. Afterwards, the overlap of the burned areas can be calculated.

It is important to notice that our methodology only displays the external boundaries of a burned area, since false negatives and unburned holes inside of burned areas could not be distinguished with simple thresholding and the available bands. This limitation needs to be considered for the different use-cases.

## Data Availability

FireSpread_MedEU is freely available at Zenodo: https://zenodo.org/records/18200075. The dataset is provided in the form of a single shapefile that contains all wildfire events and associated fire propagation steps. Additionally, pre-fire dates and dates with missing burned areas are included in the shapefile as well. It is accompanied by a pdf-file with detailed feature descriptions.

## Code availability

A code example for merging Planet swath path images, the code for the semi-automatic burned area detection and extraction, as well as the necessary images have been made available on GitHub: https://github.com/BAMresearch/wildfire_prop_database.
